# Biochemical Assessment of Bone Health in Working Obese Egyptian Females with Metabolic Syndrome; the Effect of Weight Loss by Natural Dietary Therapies

**DOI:** 10.3889/oamjms.2015.127

**Published:** 2015-12-09

**Authors:** Maha I. A. Moaty, Suzanne Fouad, Salwa M. El Shebini, Yusr I. Kazem, Salwa T. Tapozada

**Affiliations:** *National Research Centre, Nutrition and Food Sciences Department, Cairo, Egypt (Affiliation ID: 60014618)*

**Keywords:** Vitamin D, Parathyroid hormones, Bone specific alkaline phosphatase, Doum, Metabolic syndrome

## Abstract

**AIM:**

To investigate the relation between bone parameters and the metabolic syndrome criteria, before and after the administration of two different natural dietary supplements in middle aged working obese Egyptian women suffering from metabolic syndrome (MetS).

**SUBJECTS AND METHODS:**

Fifty eight middle aged obese female volunteers suffering from metabolic syndrome were divided into two groups. During the first period, group (A) consumed a low caloric diet and nutritional supplement consisting of doum flour biscuits, while group (B) consumed whole meal wheat flour biscuit with the same instructions. During the second period, both supplements were omitted. Assessment of blood pressure, relevant anthropometric parameters, lipid accumulation product, fasting blood glucose, uric acid, 25 hydroxy vitamin D (25 (OH) D), parathyroid hormone (PTH) and bone-specific alkaline phosphatase were performed.

**RESULTS:**

Data showed that although both supplements improved the MetS criteria and the bone health parameters, the supplement containing the doum flour proved to be more effective.

**CONCLUSION:**

These results confirm the benefit of doum in improving bone health parameter [25 (OH) D/PTH axis] in the MetS patients, beside the MetS criteria. So, we can conclude that natural effective supplements lead towards the optimization of biochemical parameters in favor of a healthy outcome.

## Introduction

The metabolic syndrome (MetS) is a cluster of risk factors, including abdominal obesity, hypertension, high blood glucose and triglycerides levels and low serum high density lipoprotein- cholesterol (HDL-C) concentration. The MetS might cause the development of several health problems. High levels of blood glucose are linked to many harmful changes in the body, including; changes in the kidneys’ ability to remove salt, leading to hypertension, heart disease and stroke; an increased risk of blood clot formation, which can block arteries and cause heart attacks and strokes. Uncontrolled diabetes is also associated with complications on the eyes, nerves, and kidneys [1].

The WHO defined the MetS as glucose intolerance, impaired glucose tolerance (IGT) or diabetes mellitus (DM), together with two or more of these components: hypertension, raised plasma triglycerides (≥ 150 mg/dl) and/or low HDL-C (< 35 mg/dl in men and <39 mg/dl in women), central obesity that means waist circumference (WC) ≥ 102cm (male), ≥ 88 cm (female), waist/hip ratio (WHR) > 0.90 in men and > 0.85 in women and/or body mass index (BMI) > 30 kg/m^2^, microalbuminuria, i.e., urinary albumin excretion rate ≥ 20 μg/minute or albumin/creatinine ratio ≥ 30 μg/mg, and abnormal uric acid metabolism (increased plasma uric acid concentration and/or decreased renal uric acid clearance) [2].

Vitamin D (VD) is a steroid hormone that is essential for the intestinal absorption of calcium and phosphorus, and the building of strong bones [3]. In the liver, the vitamin is hydroxylated to 25-hydroxyvitamin D (25 (OH) D), the major circulating metabolite of vitamin D. Although 1,25-dihydroxy vitamin D (1,25- (OH)_2_ D) portrays the biological active form of vitamin D, which is synthesized in the kidney, it is widely accepted that the measurement of circulating 25 (OH) D provides a better information with respect to the patients’ vitamin D status and allows its use in the diagnosis of hypovitaminosis [4].

Both of VD and parathyroid hormone (PTH) are responsible for maintaining extracellular calcium homeostasis [5]. PTH is secreted in response to low-circulating calcium concentrations. In addition to its essential role in bone health, VD has multiple extraskeletal beneficial effects as hormone regulation, inflammation reduction, and the optimal functioning of both the nervous and the immune systems [6]. Several studies in adults suggest a link between VD deficiency and cardiovascular disease risk [7], diabetes or glycosylated haemoglobin (HbA_1_c) levels [8], hypertension [9], and dyslipidemia [10]. The VD receptors are present on pancreatic β cells and insulin-sensitive tissues including skeletal muscle tissues. Reduced circulating 25 (OH) D concentrations were found to be associated with higher fasting blood glucose level, reduced insulin sensitivity and increased risk of type 2 diabetes and metabolic syndrome [11].

Bone specific alkaline phosphatase (BAP), the bone-specific isoform of alkaline phosphatase, is a glycoprotein that is found on the surface of osteoblasts, and reflects the biosynthetic activity of these bone-forming cells. BAP has been shown to be a sensitive and reliable indicator of bone metabolism [12]. Normal bone is constantly undergoing remodeling in which bone degradation or resorption is balanced by bone formation a process necessary for maintaining bone health. If the rate of resorption exceeds the rate of formation, the resulting bone loss can lead to osteoporosis and, consequently, a higher susceptibility to fractures [12, 13].

The aim of this study was to investigate the relation between the important bone health parameters and the metabolic syndrome criteria, and to verify the effect of using two dietary supplements; composed of doum in the first and whole wheat in the other, in conjunction with a balanced hypocaloric regimen in improving the MetS criteria, the 25 (OH)D/PTH axis and serum uric acid level in middle aged working obese women suffering from the metabolic syndrome.

## Material and Methods

### Materials

Wheat grains (Giza 168) was purchased from Wheat Research Department, Field Research Institute, Agriculture Research Center, Giza, Egypt. Dry doum flour was obtained from local herbal shop, Dokki, Egypt. Skimmed milk, sucrose, shortening, corn oil, baking powder, emulsifier, vanilla, bread improver and eggs were purchased from the local market, Dokki, Egypt.

### Preparation of flour

Wheat grains (Giza 168) were cleaned, tempered (15% moisture) and milled (Quadrumat Junior flour mill) to 100 % extraction flour. Whole meal wheat flour 100 % extraction (WMWF) was well blended with doum flour (DF) to produce individual mixture containing 30% replacement level. All samples were stored in airtight containers and kept at 5-7ºC until required.

### Preparation and evaluation of biscuits

Two types of biscuits recipes were prepared by using DF at 30% level with WMWF (Biscuit 1), or using WMWF alone at 100% level (Biscuit 2); to be mixed with other ingredients according to table (1). Then 14.7 ml of dextrose solution (5.93%) and a suitable amount of water were added according to AACC [14]. These formulas were baked in a special oven at 200 °C for about 15 minutes.

**Table 1:** Composition of mixtures used in the manufacture of biscuits

Ingredients (in grams)	30% DF Biscuit (1)	WMWF Biscuit (2)
Whole meal wheat flour (WMWF)	70	100
Doum flour (DF)	30	-
Corn oil	5	5
Baking powder	1.1	1.1
Salt	1.0	1.0
Emulsifier	1.0	1.0
Skimmed milk	5	5

### Subjects

Fifty eight obese female volunteers suffering from metabolic syndrome were enrolled in a program for losing weight at the nutrition department, NRC, Dokki, Gizza, Egypt. An informed consent was obtained from each participant to be included in the study. The protocol of the study was approved by the NRC Ethics committee.

Selection of the patients was based on the presence of at least three of the following five criteria of MetS according to the definition done by National Cholesterol Education Program Adult Treatment Panel III (NCEP ATP III) [15]: (1) elevated waist circumference (≥88 cm); (2) elevated blood pressure (≥130/85 mm Hg) or the use of medication for hypertension; (3) elevated fasting blood glucose: ≥100 mg/dl or the use of medication for hyperglycemia; (4) elevated serum triglycerides (TG) (≥ 150 mg/dl); (5) reduced serum HDL-C (< 50 mg/dL).

Subjects were middle aged working obese females, who had similar socioeconomic status and exposure to sunlight. None of the subjects had a history of hepatic or renal disorders, and none were taking any vitamin or mineral supplements, nor were on weight loss or exercise programs before starting the study. A detailed sheet for the diet regimen was given at the first visit to each patient; weekly follow up was performed to assure adequate compliance.

The study was divided into two phases, each one lasted for 4 weeks; the participants were divided into two groups: 34 subjects (group A), and 24 subjects (group B). During the 1^st^ phase; group (A) were consumed a low caloric diet (≈ 1000 – 1200 Kcal/day) and a nutritional supplement consisting of the doum biscuits (Biscuit 1) that was consumed as 2 biscuits at breakfast and 1 biscuit at dinner, each doum biscuit weighed 20 g and contained 30% DF, 70% WMWF. Group (B) consumed the same diet in addition to the supplement consisting of the whole wheat flour biscuit (Biscuit 2) with the same instructions. During the 2^nd^ phase, both groups were following only the low caloric balanced diet.

### Anthropometric Parameters and Blood Pressure Measurements

Relevant anthropometric measurements were reported including height, weight and minimal waist circumference (MWC) at the end of expiration, and hip circumference using the standard method [16]. Body mass index (BMI) and waist hip ratio (WHR) were calculated (weight kg/height² meter, and waist circumference cm/hip circumference cm respectively). Using Geratherm Body Fitness (B-5010), Germany, percent body fat mass (% BFM) and fat free mass (FFM) were assessed. Blood pressure was measured 3 times and the mean was recorded. The lipid accumulation product (LAP) was computed using MWC (in cm) and fasting triglycerides level (in mmol/l): [(WC – 65) × TG] (men) and [(WC – 58) × TG] (women)] [17].

### Blood Sampling and Biochemical Analysis

Blood samples were drawn in the morning from all subjects after ten hours fasting. The blood samples were allowed to clot, centrifuged and sera were separated. Fasting blood glucose (FBG) was determined in fresh sera using the oxide peroxidase method [18]. The remaining sera were divided into aliquots and stored at –70°C until used for further analysis. The fasting triglycerides were estimated enzymatically by triglycerides proceed No 2100 Stanbio Liquicolor [19]. High density lipoprotein cholesterol (HDL-C) was determined by HDL-C proceed No 0599 Stanio Liquicolor [20]. Serum 25 hydroxy vitamin D (25 (OH) D) was assessed by Vitamin D direct ELISA kit (EIA-4696) DRG ® International, Inc. USA [21]. Serum parathyroid hormone (PTH) was measured by human PTH ELISA kit (hPTH-EASIA) KAP1481 DIA source ImmunoAssays S.A. - Rue de l’Industrie, 8 - B-1400 Nivelle – Belgium [22]. BAP EIA Kit an enzyme immunoassay for the quantitation of bone-specific alkaline phosphatase (BAP) in human serum provided by MicroVue™ 8012 –Quidel Corporation 10165 McKellar Court, San Diego, CA 92121 USA [23]. Serum uric acid was determined by Uric Acid Liquicolor Test Enzymatic supplied by Stanbio [24].

All the above mentioned parameters were performed at the start of the study (1^st^ assessment), by the end of phase 1 (2^nd^ assessment) and lastly by the end of the study (3^rd^ assessment).

### Dietary Recalls

Collecting detailed basal data about nutritional habits and intake through 24hr recalls was recorded. Analysis of food items was performed using World Food Dietary Assessment System (WFDAS) 1995, USA, and University of California.

### Statistical Analysis

Statistical analyses were performed using SPSS window software version 17.0 (SPSS Inc. Chicago, IL, USA, 2008). Data were expressed as mean± SE. Two tailed Student’s t-test was used to compare between the data at the different phases. P values <0.05 were considered statistically significant. Pearson’s correlation coefficient (r) was calculated to find the associations between different variables.

## Results

Data presented in Table 2 showed the composition of the prepared biscuits. Protein, fat and total carbohydrate contents were lower in biscuits substituted with DF compared to the biscuit prepared from WMWF alone. Addition of DF to WMWF caused changes in the mineral contents of biscuits. Polyphenol content of the DF biscuit was more than of the WMWF biscuit.

**Table 2:** Nutrient contents of the two types of the biscuits

Constituents	30% DF Biscuit	WMWF Biscuit (0% DF)
Moisture (%)	4.80 ± 0.12	3.82± 0.22
Protein (g)	11.75 ± 0.34	13.5 ± 0.18
Fat (g)	6.50 ± 0.15	7.25 ± 0.35
Carbohydrates (g)	72.45 ± 0.69	75.62 ± 0.77
Crude fiber (g)	5.60 ± 0.40	1.85 ± 0.02
Total Ash (g)	3.70 ± 0.25	1.78 ± 0.12
Phosphorus (mg)	305 ± 0.11	187± 0.12
Potassium (mg)	290 ± 0.21	115± 0.09
Calcium (mg)	70 ± 0.01	40± 0.03
Magnesium (mg)	84 ± 0.03	115±0.08
Sodium (mg)	420 ± 0.13	650± 0.11
Iron (mg)	2.8 ± 0.01	3.5±0.03
Polyphenols (mg)	164.3	110.0

Values are means of three determinations ± SE.

Table 3 & 4 showed a comparison between the different macronutrients and micronutrients and the percent caloric distribution of the habitual diet of the whole sample before starting the regimen and of the two types of regimen.

**Table 3:** Mean ± SE of the daily nutrients intake of habitual diet in the whole sample, and of the two different regimens

Nutrients	Habitual diet	Regimen with 30%DF Biscuits	Regimen with WMWF Biscuits
Energy (kcal)	2230.02 ± 9.18	1127.18 ± 7.21	1143.39 ± 4.16
Protein (g)	89.74 ± 4.59	1127.18 ± 7.21	58.02 ± 2.33
Carbohydrate (g)	265.90 ± 12.88	142.33 ± 6.22	144.0 ± 6.28
Fat (g)	87.19 ± 4.81	34.421 ± 2.14	34.87 ± 1.64
Calcium (mg)	765.37 ± 6.20	537.21 ± 5.22	519.21 ± 3.25
Iron (mg)	16.69 ± 0.97	12.82 ± 1.42	13.24 ± 0.12
Sodium (mg)	3919.55 ± 2.35	1985.40 ± 3.50	2123.46 ± 3.47
Potassium (mg)	2871.25 ± 5.56	2947.14 ± 3.43	2842.14 ± 3.18

**Table 4:** Percent caloric intake from the three main macronutrients of the habitual diet in the whole sample, and of the two different regimens

	Habitual diet	Regimen with 30% DF	Regimen with WMWF Biscuits
Total Calories	2230.02	1127.2	1143.4
% Calories from protein	16.1	20.2	20.3
% Calories from carbohydrate	47.7	50.4	50.4
% Calories from fat	35.2	27.5	27.4

The data showed the balanced and healthy distribution of the macronutrients in the two regimens compared to the habitual diet of the patients.

Table 5 showed that at the end of 1^st^ phase of the study (2^nd^ assessment), there was a highly significant (p < 0.01) decrease of % body fat mass (%BFM), mean minimal waist circumference (MWC) and waist hip ratio (WHR) in both groups, but % decrease was greater in the doum group (group A) compared with the whole wheat group (group B). At the end of the 2^nd^ phase of the study (3^rd^ assessment), patients were on the low caloric diet alone, % BFM, FFM, MWC and WHR showed highly significant decrease (p < 0.01) in group (A), while in group (B) only MWC was significantly decreased (p < 0.05). SBP, DBP and LAP of both groups decreased significantly by the end of 1^st^ phase (2^nd^ assessment), only DBP of group B decreased significantly at the end of 2^nd^ phase (3^rd^ assessment), while other reading values decreased numerically except LAP of group B which increased numerically.

**Table 5:** The effect of the two dietary supplements on clinical and anthropometric parameters, and metabolic syndrome criteria

Parameters	Group A (no.= 34 Subjects)			Group B (no.= 24 Subjects)		

	1^st^assessment	2^nd^assessment	3^rd^assessment	1^st^assessment	2^nd^assessment	3^rd^assessment
Age(yr)	50.3±0.81			46.9±0.87		

SBP (mmHg)	137.7±1.91	131.9±2.40^*a^	128.4±2.85	138.2±3.27	125.8±3.04^**a^	123.6±2.51

% change		-4.2	-2.7	-	-8.9	-1.7

DBP (mmHg)	91.3±1.42	86.4±1.17^**a^	83.9±1.04	89.0±1.75	84.6±1.44^**a^	82.3±1.64^*b^

% change		-5.4	-2.9	-	-4.9	-2.7

BMI (kg/m^2^)	38.8±1.24	37.6±1.23^**a^	36.6±1.44^**b^	38.2±1.33	37.1±1.22^**a^	36.8±1.27

% change		-3.9	-2.7	-	-2.9	-0.8

Body fat mass%	45.37 ±2.29	43.14 ±2.29^xxa^	41.17 ±2.67^**b^	44.77 ±2.51	42.72 ±2.33^**a^	42.23 ±2.43

% change		-4.9	-4.6	-	-4.6	-1.1

Fat free mass	51.38 ±1.07	50.61 ±1.08^**a^	49.68 ±1.22^xxb^	51.77 ±1.33	50.89 ±1.29^**a^	50.87 ±1.37

% change		-1.5	-1.8	-	-1.7	-0.03

MWC (cm)	101.9 ±2.12	97.0 ±2.00^**a^	92.9 ±2.55^**b^	96.1 ±2.05	92.1 ±1.99^**a^	90.7 ±1.88^*b^

% change		-4.8	-4.2	-	-4.2	-1.5

WHR (cm/cm)	0.83 ±0.01	0.81 ±0.01^**a^	0.78 ±0.01^**b^	0.79 ±0.01	0.78 ±0.01^**a^	0.76 ±0.01

% change		-2.4	-3.7	-	-1.3	-2.6

Hip (cm)	123.6 ±2.12	120.0 ±1.98^**a^	118.2 ±2.45^**b^	122.1 ±2.33	118.8 ±2.27^**a^	116.9 ±2.21^**b^

% change		-2.9	-1.5	-	-2.7	-1.6

BMR	2406	2368	2332	2402	2368	2359

Lipid accumulation product	8316.0 ± 880.46	5811.8 ±721.67^**a^	5250.9 ±698.79	5168.8 ±546.81	3638.9 ±287.28^xxa^	3904.28 ±269.83

% change		-30.1	-9.7	-	-29.6	+7.3

FBG (mg/dl)	128.9±7.14	106.9±6.06^**a^	111.3±5.67	106.3±7.13	89.5±3.10^*a^	92.7±2.16

% change		-17.1	4.2		-15.8	3.6

TG (mg/dl)	188.5±13.68	149.0±11.68^**a^	156.2±13.88	136.0±8.64	107.1±6.71^**a^	122.6±8.51^*b^

% change		-20.5	4.2		-21.3	14.5

HDL-C (mg/dl)	47.1±1.06	59.6±1.44^**a^	55.5±2.45^*b^	43.9±2.01	54.2±2.05^**a^	49.5±1.82^*b^

% change		26.5	-6.9		23.5	-8.7

SBP: Systolic blood pressure, DBP: Diastolic blood pressure, BMI: Body mass index, MWC: Minimal waist circumference, WHR: waist hip ratio, BMR: Basal metabolic rate, FBG: Fasting blood glucose, TG: Triglycerides, HDL-C: High density lipoprotein cholesterol.

* P value < 0.05,

** P value < 0.01

a 1^st^ assessment vs. 2^nd^ assessment

b 2^nd^ assessment vs. 3^rd^ assessment

At the 2^nd^ assessment the mean levels of the fasting blood glucose (FBG) and triglyceride (TG) decreased significantly (p 0.05 - < 0.01), while the high density lipoprotein–cholesterol (HDL-C) concentration increased significantly, in both groups. The 3^rd^ assessment showed elevation of both FBG and TG, and reduction of the HDL-C levels.

The biochemical parameters of the participants at the three different assessments are shown in Table 6. At the 1^st^ assessment, 25 (OH) D level was in the range considered to be deficient (≤ 50 nmol/L) or insufficient (≤ 75 nmol/L), however the mean PTH level was higher than the normal range in both groups (normal PTH ≤ 65 pg/ml).

**Table 6:** Mean ± SE of the biochemical parameters of the two groups at the basal and the two phases of the study

Parameters	Group A (no.= 34 Subjects)			Group B (no.=24 Subjects)		

	1^st^assess-ment	2^nd^assess-ment	3^rd^assess-ment	1^st^ assess-ment	2^nd^assess-ment	3^rd^assess-ment
25 (OH) D (nmol/L)	51.9 ±2.2	56.2 ±2.3^**a^	57.6 ±3.3^*b^	48.1 ±1.7	51.3 ±1.8^*a^	53.8 ±1.7^*b^

% change		+8.3^**a^	+2.5^*b^	-	+6.7^*a^	+4.9^*b^

PTH (pg/ml)	81.2 ±2.8	77.3±2.5^**a^	80.6 ±2.5	84.2 ±3.2	80.8 ±2.9^*a^	81.7 ±2.7

% change		-4.8^**a^	+4.3	-	-4.04	+1.11

BAP (µg/L)	25.4 ±1.3	24.3±1.4	24.4 ±1.4	25.2 ±1.3	23.0 ±1.3^*a^	23.4 ±1.3

% change		-4.3	+0.4	-	-8.7^*a^	+1.7

Uric acid (mg/dl)	5.9 ±0.2	5.1 ±0.1^**a^	4.9 ±0.1	5.6 ±0.2	4.8 ±0.2^**a^	4.5 ±0.2

% change		-13.6^**a^	-3.9	-	-14.3^**a^	-4.2

25 (OH) D: 25 hydroxy vitamin D, PTH: Parathyroid hormone, BAP: Bone specific alkaline phosphatase.

* P value < 0.05

** P value < 0.01;

a 1^st^ assessment vs. 2^nd^ assessment,

b 2^nd^ assessment vs. 3^rd^ assessment.

After dietary supplement consumption, 25 (OH) D was significantly increased in both groups (p < 0.01 in group A, p < 0.05 in group B), while the mean level of the PTH was significantly decreased in both groups. At the 3^rd^ assessment, 25 (OH) D increased significantly in both groups (p < 0.05), while the mean levels of the PTH were numerically increased. The mean levels of the BAP were elevated at the 1^st^ assessment in both groups and decreased numerically with weight loss in group (A), but significantly decreased in group (B) at p < 0.05. In the 2^nd^ phase of the studied BAP levels increased without showed statistical significance. Uric acid levels were significantly decreased in both groups after dietary supplement but the % change of decrease was greater in group (B) as compared with group (A).

Table 7 showed the correlation coefficient between the different studied parameters. The most important significant negative correlation was found between PTH and 25 (OH) D in both groups. The 25 (OH) D showed significant positive correlation with HDL-C in group (A), and negative correlation with DBP and TG in group (B).

**Table 7:** Correlation coefficient between the bone health parameters and the metabolic syndrome criteria at the basal and the two phases of the study

	BMI	MWC	SBP	DBP	FBG	TG	HDL-C	25 (OH) D	BAP
Group A Parathyroid hormone (pg/ml)									

1^st^ assessment	0.498^^**^^	0.313	0.069	0.062	0.202	0.452^^**^^	0.135	-0.601^^**^^	0.184

2^nd^ assessment	0.429^*^	0.298	0.142	0.286	0.179	0.287	-0.360^*^	-0.691^^**^^	0.314

3^rd^ assessment	0.564^^**^^	0.469^^**^^	0.032	0.199	0.318	0.604^^**^^	0.003	-0.709^^**^^	0.118

Group B Parathyroid hormone (pg/ml)									

1^st^ assessment	0.075	0.201	0.439^*^	0.487^*^	0.299	0.359	0.211	-0.519^^**^^	0.459^*^

2^nd^ assessment	0.198	0.247	0.446^*^	0.652^^**^^	0.126	0.104	0.204	-0.719^^**^^	0.145

3^rd^ assessment	0.114	0.291	0.678^^**^^	0.617^**^	0.116	0.292	-0.067	-0.647^^**^^	0.094

Group A 25 hydroxy vitamin D (nmol/L)									

1^st^ assessment	-0.196	-0.103	0.283	0.236	-0.247	-0.241	0.185	-	-0.211

2^nd^ assessment	-0.208	-0.106	-0.124	0.163	-0.286	-0.113	0.543^**^	-	-0.041

3^rd^ assessment	-0.207	-0.123	0.033	-0.024	-0.141	-0.320	0.210	-	-0.126

Group B 25 hydroxy vitamin D (nmol/L)									

1^st^ assessment	0.302	0.087	0.032	-0.134	0.351	0.146	0.340	-	0.053

2^nd^ assessment	-0.298	-0.340	-0.242	-0.577^^**^^	0.081	-0.549^^**^^	0.071	-	-0.162

3^rd^ assessment	-0.214	-0.289	-0.309	-0.487^*^	-0.196	-0.502^*^	0.046	-	-0.185

Group A Bone specific alkaline phosphatase (μg/L)									

1^st^ assessment	0.094	0.005	0.321	0.369^*^	0.154	0.006	-0.290	-0.211	-

2^nd^ assessment	0.210	0.092	0.175	0.014	0.062	0.088	-0.249	-0.041	-

3^rd^ assessment	0.146	0.119	0.036	0.185	0.241	0.291	-0.050	-0.126	-

Group B Bone specific alkaline phosphatase (μg/L)									

1^st^ assessment	0.070	0.089	0.283	0.428^*^	0.153	0.231	0.130	0.053	-

2^nd^ assessment	0.115	0.076	0.310	0.210	0.410^*^	0.066	0.302	-0.162	-

3^rd^ assessment	0.238	0.168	0.237	0.115	-0.157	0.072	0.176	-0.185	-

BMI: Body mass index, MWC: Minimal waist circumference, SBP: Systolic blood pressure, DBP: Diastolic blood pressure, FBG: Fasting blood glucose, TG: Triglycerides, HDL-C: High density lipoprotein cholesterol. Numbers presented in this table are the value of r =correlation coefficient.

* Correlation is significant at the 0.05 level (2-tailed)

** correlation is significant at the 0.01 level (2- tailed).

There was a significant positive correlation between PTH and BMI, MWC and TG in group (A), while in group (B), PTH was significantly positively correlated with SBP, DBP and BAP. FBG and BAP were positively correlated in group B. Non significant negative correlation was found between 25 (OH) D and BAP levels.

## Discussion

The tenet “Let food be the medicine and medicine be the food,” espoused by Hippocrates nearly 2,500 years ago, is receiving renewed interest. In particular, there has been an explosion of consumer interest in the health enhancing role of specific foods or physiologically-active food components, so-called functional foods [25]. Functional foods either from plant or animal sources may enhance health [26].

Doum palm *(Hyphaene thebaica)* is a desert palm native to Egypt. It is known in Egypt as the doum or gingerbread palm which grows to a height of 6 or 9 m and usually has forked stems with fan shaped leaves, 65–75 cm long. It is listed as one of the useful plants of the world [27].

The data in this study revealed that patients who consumed biscuits made from the 30% DF and biscuit with WMWF showed significant decreases in their anthropometric parameters. Furthermore, chemical analysis results revealed that adding 30% DF to the WMWF biscuits increased the fiber and the polyphenols contents of the biscuits. The influence of these components was especially observed on the BMI and MWC which showed the higher percent decrease among all the anthropometric parameters in both groups, yet group (A) who consumed 30% DF biscuits showed the higher decrease when compared to group (B). In this context visceral obesity may represent a clinical intermediate phenotype reflecting the relative inability of subcutaneous adipose tissue to act as a protective metabolic sink for the clearance and storage of the extra energy derived from dietary triglycerides, leading to ectopic fat deposition in visceral adipose depots [28].

The results of this study demonstrated the effect of the two dietary supplements on LAP and blood pressure measurements more than the effect of low caloric diet alone. In view of the role of central obesity and dyslipidemia in the atherosclerotic process, an alternative continuous index of lipid over accumulation, LAP has been proposed; LAP is a simple indicator that requires only the determination of circulating triglycerides and measurement of MWC. The MWC is unable to distinguish between visceral adipose tissue and subcutaneous adipose tissue. Visceral adiposity is more strongly associated with cardiometabolic risks compared with subcutaneous adipose tissue [17]. Visceral adipose tissue adipocytes have a higher rate of lipolysis and also produce more adipocytokines, such as interleukin-6 and plasminogen activator inhibitor-1 [29]. Therefore, it is important to include a routinely applicable indicator for evaluation of visceral adiposity. Triglycerides have been reported as a significant correlate with visceral adipose tissue in healthy men, even after controlling for abdominal subcutaneous adipose tissue. LAP will increase as more lipids are deposited in non-adipose “ectopic” tissues such as the liver, blood vessels, pancreas, kidneys and skeletal muscles, where they may adversely affect cellular function and interfere with cardiovascular regulation [17].

The baseline data demonstrated 25 (OH) D deficiencies. Dietary intervention improved the vitamin level that coincided with weight reduction especially with decreased BMI, %BFM, FFM, and WHR. The basis of low 25 (OH) D concentrations in obesity is still under debate and could be the result of several mechanisms. One hypothesis is that the high content of body fat acts as a reservoir for lipid soluble VD and increases its sequestration, thus determining its low bioavailability [30]. In obese subjects, not only fat mass is increased but also lean body mass, as an adaptive response to greater body weight. In animal studies it has been shown that VD was stored 33% in fat and 20% in muscle [31], suggesting that muscle could be also another reservoir of VD in humans. Another hypothesis is that the synthesis of 25 (OH) D by the liver may occur at a lower rate in obese subjects due to hepatic steatosis [32]. An alternative explanation is that higher leptin and interleukin 6 circulating levels, mostly secreted by adipose tissue, and may have inhibitory effects on 25 (OH) D syntheses via their receptors [33].

Several weight loss/diet interventions have measured VD before and after weight loss, and observed that 10% loss in weight and % BFM, and 9 % reduction in MWC resulted in a greater increase in 25 (OH) D levels (about 34 %). Yet the study suggested there might be a threshold of weight loss or time needed to see a significant increase in 25 (OH) D levels. However, other researchers found a significant seasonal variation in 25 (OH) D concentrations than changes in weight or fat mass. The researchers found a significant season by time interaction, indicating that the change in 25 (OH) D levels was dependent on season during enrolment and suggested seasonal variation may have had a greater impact on 25 (OH) D than changes in weight or fat mass [34].

The PTH concentrations were elevated in our study in concordance with several previous studies in adults. It has long been accepted that both depressed 25-(OH) D and reactive rises in PTH were consequences of obesity. It is also plausible that depressed 25 (OH) D and elevated PTH levels might also play a role in the development of obesity as there are known physiological mechanisms through which depressed 25 (OH) D and/or PTH elevations promote the accumulation of adipose tissue [35]. VD has been postulated to mediate the effect of PTH on intracellular calcium influx as a mechanism for increased fat storage [36]. In this study, the mean BAP levels were elevated which was associated with the detected PTH elevation. However previous studies also have shown that postmenopausal women have higher BAP levels than do premenopausal subjects and that this may be due to the increased bone loss observed after menopause [37].

Previous studies have showen a close relation between hyperuricemia and metabolic syndrome in adults [38]. Although hyperuricemia is well recognized as a risk factor for atherosclerotic diseases such as myocardial infarction and stroke, the independence of this association from other confounding factors has remained controversial. This is mostly because serum uric acid is associated with other cardiovascular risk factors, such as hypertension and dyslipidemia [39]. However, Makovey et al. [40], reported that higher serum uric acid levels appear to be protective for bone loss in peri- and postmenopausal women as being a strong endogenous antioxidant, and this relationship is not affected by changes in body composition measures. Data of this study showed that the serum uric acid concentration among the patients at the baseline assessment was at the high normal range, and then a significant reduction was observed after the dietary intervention, less reduction was detected after stopping the supplement, and that is where the use of the supplements that contain antioxidant ingredients, is likely to have led to a lack of the body’s need to raise the proportion of uric acid as an antioxidant, to avoid the other pathological diseases caused by the high uric acid concentration as already mentioned.

In conclusion, using a dietary therapy composed of a hypocaloric balanced regimen and a supplement formulae made from doum flour and whole meal wheat flour, resulted in improving the 25(OH) D/PTH axis by increasing the level of 25(OH) D and decreased parathyroid hormone, which lead to normalization of the bone specific alkaline phosphatase, that can promote and protect bone health. Decreasing uric acid and lipid accumulation product is an additional benefit which could potentially support metabolic syndrome obese patients.

## References

[1] Mottillo S, Filion KB, Genest J (2010). The metabolic syndrome and cardiovascular risk a systematic review and meta-analysis. J Am Coll Cardiol.

[2] Alberti K, Zimmet T (2013). “Definition, Diagnosis, and Classification of Diabetes Mellitus and its Complications”. World Health Organization.

[3] Lips P (2010). Worldwide status of vitamin D nutrition. J Steroid Biochem Mol Biol.

[4] Wielders JP, Wijnberg FA (2009). Preanalytical stability of 25 (OH) vitamin D3 in human blood or serum at room temperature: solid as a rock. Clin Chem.

[5] Soares MJ, Murhadi LL, Kurpad AV (2012). Mechanistic roles for calcium and vitamin D in the regulation of body weight. Obes Rev.

[6] Grethen E, McClintock R, Gupta CE (2011). Vitamin D and hyperparathyroidism in obesity. Journal of Clinical Endocrinology and Metabolism.

[7] Ku YC, Liu ME, Ku CS (2013). Relationship between vitamin D deficiency and cardiovascular disease. World Journal of Cardiology.

[8] Kayaniyil S, Vieth R, Retnakaran R (2010). Association of vitamin D with insulin resistance and beta-cell dysfunction in subjects at risk for type 2 diabetes. Diabetes Care.

[9] Pittas AG, Chung M, Trikalinos T (2010). Systematic review: vitamin D and cardiometabolic outcomes. Annals of Internal Medicine.

[10] Karhapää P, Pihlajamäki J, Pörsti I (2010). Diverse associations of 25-hydroxyvitamin D and 1,25-dihydroxy-vitamin D with dyslipidaemias. J Intern Med.

[11] Vimaleswaran KS, Berry DJ, Lu C (2013). Causal relationship between obesity and vitamin D status: bi-directional Mendelian randomization analysis of multiple cohorts. PloS Medicine.

[12] Kress BC (1998). Bone alkaline phosphatase: methods of quantitation and clinical utility. J Clin Ligand Assay.

[13] Kress BC, Mizrahi IA, Armour KW (1999). Use of bone alkaline phosphatase to monitor alendronate therapy in individual postmenopausal osteoporotic women. Clin Chem.

[14] AOAC (2000). Official Methods of Analysis. Association of Official Analytical Chemist. EUA.

[15] Gupta A, Gupta V (2010). Metabolic syndrome: what are the risks for humans?. Biosci Trends.

[16] Tanner JM, Hiernau J, Jerman S, Weiner JS, Lourie SA (1969). Growth and physical studies In Human Biology: A guide to field methods.

[17] Taverna MJ, Martínez-Larrad MT, Frechtel GD (2011). Lipid accumulation product: a powerful marker of metabolic syndrome in healthy population. Eur J Endocrinol.

[18] Barham D, Trinder P (1972). An improved color reagent for determination of blood glucose by oxidase system. Analyst.

[19] Allain CC, Poon LS, Chan CS, Richmond W, Fu PC (2011). Enzymatic determination of total serum cholesterol. Clin Chemo.

[20] Wornick DF, Albers JJ (1978). A comprehensive evaluation of the heparin-manganese precipitation procedure for estimating high density lipoprotein cholesterol. J Lipid Res.

[21] Wielders JP, Wijnberg FA (2009). Preanalytical stability of 25(OH)-vitamin D3 in human blood or serum at room temperature: solid as a rock. Clin Chem.

[22] Bouillon R, Coopmans W, De Groote D (1990). Immunoradiometric assay of Parathyrin with polyclonal and monoclonal region specific antibodies. Clin Chem.

[23] Garnero P, Delmas PD, Meunier PJ (1998). Clinical usefulness of markers of bone remodeling in osteoporosis. Osteoporosis: Diagnosis and management.

[24] Fossati P, Prencipe L, Berti G (1980). Use of 3, 5-Dichloro-2-hydroxy benzene sulfonic Acid/4-Ami nophenazone chromogenic system in direct enzymatic assay of uric acid in serum and urine. Clin Chem.

[25] Hasler CM (1998). A new look at an ancient concept. Chem. Industry Feb.

[26] Abdel-Moaty M, Fouad S, El-Shebini S (2015). Serum Ceramide Kinase as a Biomarker of Cognitive Functions, and the Effect of Using Two Slimming Dietary Therapies in Obese Middle Aged Females. OA Maced J Med Sci.

[27] Abdel-Moaty M, El-Shebini S, Ahmed N (2014). Serum Level of the Adipokine “Vaspin” in Relation to Metabolic Parameters: Short - Term Effect of Specific Dietary Therapy. Maced J Med Sci.

[28] Després J.P.I, Lemieux J, Bergeron P (2008). Abdominal obesity and the metabolic syndrome: contribution to global cardiovascular risk. Arterioscler Thromb Vasc Biol.

[29] Hamdy O, Porramatikul S, Al-Ozairi E (2006). Metabolic obesity: the paradox between visceral and subcutaneous fat. Curr Diabetes Rev.

[30] Wortsman J, Matsuoka L, Chen T (2000). Decreased bioavailability of vitamin D in obesity. American Journal Clinical Nutrition.

[31] Mawer E, Backhouse J, Holman C (1972). The distribution and storage of vitamin D and its metabolites in human tissues. Clinical Science.

[32] Targher G, Bertolini L, Scala L (2007). Associations between serum 25-hydroxyvitamin D3 concentrations and liver histology in patients with non-alcoholic fatty liver disease. Nutrition, Metabolism and Cardiovascular Diseases.

[33] Ding C, Parameswaran V, Blizzard L (2010). Not a simple fat-soluble vitamin: changes in serum 25-(OH)D levels are predicted by adiposity and adipocytokines in older adults. Journal of Internal Medicine.

[34] Tzotzas T, Papadopoulou FG, Tziomalos K (2010). Rising serum 25-hydroxy-vitamin D levels after weight loss in obese women correlate with improvement in insulin resistance. J Clin Endocrinol Metab.

[35] Snijder MB, van Dam RM, Visser M (2005). Adiposity in relation to vitamin D status and parathyroid hormone levels: a population-based study in older men and women. Journal of Clinical Endocrinology and Metabolism.

[36] Portale AA, Miller WL (2000). Human 25-hydroxylation D-1ahydroxylase: cloning, mutations, and gene expression. Pediatric Nephrology.

[37] Schiele F, Henny J, Hitz J (1983). Total bone and liver alkaline phosphatase in plasma: biological variations and reference limits. Clin Chem.

[38] Ishizaka N, Ishizaka Y, Toda E (2005). Association between serum uric acid, metabolic syndrome, and carotid atherosclerosis in Japanese individuals. Arterioscler Thromb Vasc Biol.

[39] Castrop H (2007). Mediators of tubuloglomerular feedback regulation of glomerular filtration: ATP and adenosine. Acta Physiol (Oxf).

[40] Makovey J, Macara M, Chen JS, Hayward CS, March L, Seibel MJ, Sambrook PN (2013). Serum uric acid plays a protective role for bone loss in peri- and postmenopausal women: a longitudinal study. Bone.

